# mTOR independent regulation of macroautophagy by Leucine Rich Repeat Kinase 2 via Beclin-1

**DOI:** 10.1038/srep35106

**Published:** 2016-10-12

**Authors:** Claudia Manzoni, Adamantios Mamais, Dorien A. Roosen, Sybille Dihanich, Marc P. M. Soutar, Helene Plun-Favreau, Rina Bandopadhyay, John Hardy, Sharon A. Tooze, Mark R. Cookson, Patrick A. Lewis

**Affiliations:** 1 School of Pharmacy, University of Reading, Whiteknights, Reading, RG6 6AP, United Kingdom; 2 Department of Molecular Neuroscience, UCL Institute of Neurology, Queen Square, London WC1N 3BG, United Kingdom; 3 Cell Biology and Gene Expression Section, Laboratory of Neurogenetics, NIA, NIH, Building 35, 35 Convent Drive, Bethesda, MD 20892-3707, USA; 4 Reta Lila Weston Institute of Neurological Studies, UCL Institute of Neurology, 1 Wakefield Street London WC1N 1PJ, United Kingdom; 5 Francis Crick Institute, London Research Institute, 44 Lincoln’s Inn Fields London, WC2A 3LY, United Kingdom

## Abstract

Leucine rich repeat kinase 2 is a complex enzyme with both kinase and GTPase activities, closely linked to the pathogenesis of several human disorders including Parkinson’s disease, Crohn’s disease, leprosy and cancer. LRRK2 has been implicated in numerous cellular processes; however its physiological function remains unclear. Recent reports suggest that LRRK2 can act to regulate the cellular catabolic process of macroautophagy, although the precise mechanism whereby this occurs has not been identified. To investigate the signalling events through which LRRK2 acts to influence macroautophagy, the mammalian target of rapamycin (mTOR)/Unc-51-like kinase 1 (ULK1) and Beclin-1/phosphatidylinositol 3-kinase (PI3K) pathways were evaluated in astrocytic cell models in the presence and absence of LRRK2 kinase inhibitors. Chemical inhibition of LRRK2 kinase activity resulted in the stimulation of macroautophagy in a non-canonical fashion, independent of mTOR and ULK1, but dependent upon the activation of Beclin 1-containing class III PI3-kinase.

Leucine rich repeat kinase 2 is one of the key genetic factors contributing to the risk of developing Parkinson’s disease (PD), an irreversible, progressive neurodegenerative movement disorder primarily associated with neuronal cell loss in the *Substantia nigra pars compacta*. Coding mutations in the *LRRK2* gene are the most frequent genetic cause of familial PD, with polymorphisms in *LRRK2* associated with an increased risk of idiopathic PD^1^,^2^,^3^,^4^. In addition to this, genome wide association (GWA) studies recently identified the *LRRK2* locus as being involved in the risk for PD^5^, Crohn’s disease^6^ and multibacillary leprosy^7^,^8^. Mutations in LRRK2 have also been linked to cancer^9^, and the *LRRK2* region was identified as being subject to frequent carcinogenic alterations^10^. The *LRRK2* gene is therefore related to the etiopathogenesis of at least four human diseases, making it the focus of increasing attention as a putative drug target.

The physiological function of LRRK2 is as yet unclear. It is a complex enzyme, with active kinase and GTPase domains that are thought to reciprocally regulate one another’s activity^11^,^12^. As detailed in the following section, several studies have indicated a putative role for LRRK2 in the control of macroautophagy, a process used by the cell to maintain a healthy microenvironment by removing misfolded proteins and damaged organelles^13^. The molecular mechanism underlying this association remains to be fully understood. While LRRK2 over-expression was associated with a macroautophagy-dependent induction of toxicity coupled with neurite atrophy^14^, LRRK2 knock down was shown to both reduce and potentiate the autophagic flux^15^,^16^. Moreover, the overexpression of full-length LRRK2, or its kinase domain, as well as inhibition of LRRK2 kinase activity induced alterations of the macroautophagy-lysosomal pathway^17^,^18^,^19^. Macroautophagy was shown to be altered in human fibroblasts carrying LRRK2 pathogenic mutations associated with PD^20^,^21^, in neurons derived from those human fibroblasts^22^ and in transgenic or LRRK2 knock-out mouse models^23^. Finally, pathogenic mutations in LRRK2 have been linked to deregulation of chaperone mediated autophagy (CMA)^24^. More generally, LRRK2 was associated with vesicle trafficking and synaptic functionality^25^,^26^, and with endocytosis and trans-Golgi network homeostasis^27^,^28^. A hypothetical function for LRRK2 in the regulation of macroautophagy, and in general in vesicle homeostasis, is compelling considering that the macroautophagy/lysosomal system has an increasingly appreciated link to the etiology of PD^29^, while it has long been considered a central player in the pathogenesis of Crohn’s, leprosy and cancer.

The data presented herein demonstrate that the kinase activity of LRRK2 acts as a negative regulator of macroautophagy in astrocyte cell models. Our results suggest that LRRK2 may act to control a non-canonical pathway alternative and parallel to that regulated by the mammalian target of rapamycin (mTOR) and Unc-51 Like Kinase 1 (ULK1), but dependent on the presence of an active Beclin-1 complex. These data have important implications for the study of the physiological and pathological functions of LRRK2, in particular for any pharmacological intervention based upon LRRK2 inhibition.

## Results

### Inhibition of LRRK2 kinase activity increases LC3-II levels

**Figure 1:**
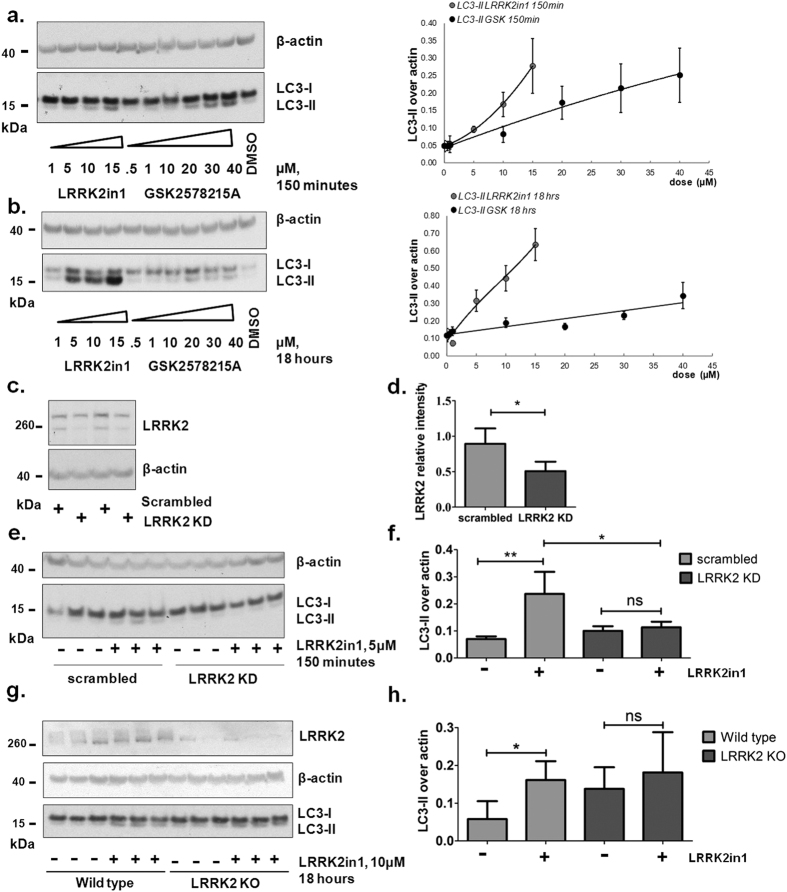
Inhibition of LRRK2 kinase leads to a dose response and time dependent increase in LC3-II. 150 minutes (**a**) or 18 hours (**b**) LRRK2in1 and GSK2578215A dose-response. The gels shown are representative of 3 independent experiments, LC3-II was quantified against β-actin, quantification was done for each single experiment; after normalization against the control in DMSO, data were pooled together in the dose-response curve (mean and SEM). (**c**) H4 cells with stable LRRK2 knock-down (~50% LRRK2 knock-down for KD#644167.03b transfection) as quantified in (**d**); LRRK2 was quantified (sum of the upper and lower bands) against β-actin, statistical analysis was performed by unpaired student t-test (mean and SD, p value = 0.0236). (**e**) 150 minutes LRRK2in1 treatment in scramble controls and in LRRK2 knock-down cells; the image shown (reporting 3 samples for each condition) is representative of 3 independent experiments and is quantified in (**f**); LC3-II was quantified against β-actin; statistical analysis was performed by 1way Anova followed by Tukey post-hoc test (mean and SD, **p < 0.01; *p < 0.05). (**g**) primary astrocytes from wild-type and LRRK2 knock-out mice treated with LRRK2in1 for 18 hours, the image shown is representative of 3 independent cell preparations, each replicated with 3 samples. (**h**) LC3-II was quantified against β-actin for each single experiment; after normalization against the internal control in DMSO, data were pulled together and statistical analysis was performed by 1way Anova followed by Tukey post-hoc test. (mean and SD, *p < 0.05).

LRRK2 is expressed at high levels in astrocytes within the brain^30^,^31^. Human H4 neuroglioma cells, originally derived from an astrocytoma, were previously used as a model to study LRRK2 function in macroautophagy^18^,^30^. Based on a previous work by our group^18^, we here replicate and expand our previous analysis confirming that treatment of H4 cells for 150 minutes (acute treatment) or 18 hours (chronic treatment) with LRRK2 kinase inhibitors, either LRRK2in1^32^ or GSK2578215A^33^ result in a concentration dependent increase of LC3-II (Fig. 1a,b); no concomitant toxicity was recorded for the LRRK2in1 while a decrease in cell survival was detected for GSK2578215A starting at 30 μM (Supplementary Fig. S1a,b)^34^. A major confounding factor when using chemical inhibitors of enzymes is the possibility of off target effects. Although the inhibitors used are structurally distinct, it is critical to demonstrate that the cellular phenotypes measured are specific to the protein of interest. To achieve this, and as already previously proposed by our group, endogenous LRRK2 protein levels in H4 cells were decreased (~50%) by stable expression of LRRK2 shRNA (Fig.1c,d). 150 minutes (Fig. 1e,f) or 18 hours (Supplementary Fig. S1c) inhibition of LRRK2 kinase activity by LRRK2in1 increased LC3-II in scrambled controls cells but not in LRRK2 knocked-down cells, strongly suggesting that this is a LRRK2 dependent phenomenon. Interestingly, in this model system we consistently see no increase in basal LC3-II when knockin-down LRRK2. Further investigations are needed, however we suggest this may happen either because the knock-down is never complete, or alternative splicing isoforms may originate. Moreover, it is worth noticing that, with the knock-down strategy, we remove the kinase as well as the GTPase activities and the protein-protein interaction domains of LRRK2. Therefore the chemical kinase inhibition and the protein knock-down approaches may not represent a perfect phenocopy of each other. Primary astrocytes from LRRK2 knock-out mice were prepared and assessed for response to LRRK2 inhibition. An elevated, basal level of LC3-II was detected in the knock-out cells that was not significantly increased following treatment with LRRK2in1 (Fig. 1g,h, see Supplementary Fig. S1e for astrocytes evaluation by GFAP staining).

### LRRK2 dependent increase of LC3-II is not due to decreased autophagosome-lysosome fusion

**Figure 2:**
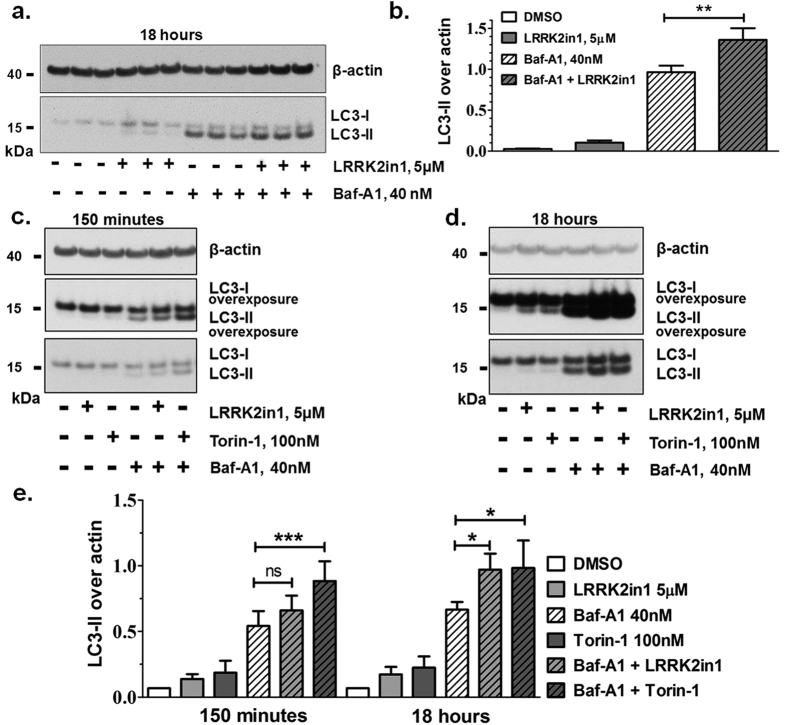
Increase in LC3-II after inhibition of LRRK2 kinase is due to an induction of the macroautophagy flux. (**a**) 18 hours treatment with LRRK2in1 in the presence and absence of BafA1 to block the autophagy flux. The gel shown is representative of 3 independent experiments, each performed in triplicate and it is quantified in (**b**). (**b**) LC3-II is quantified against β-actin; statistical analysis was performed by 1way Anova followed by Tukey post-hoc test (mean and SD, **p < 0.01). 150 minutes (**c**) and 18 hours (**d**) treatment with LRRK2in1 or Torin-1 (to induce macroautophagy) in the presence and absence of BafA1. The gels shown are representative of at least 4 independent experiments summarized in (**e**). (**e**) LC3-II is quantified against β-actin for 5 replicates (150 minutes) and 4 replicates (18 hours); quantification was done for each single experiment; after normalization against the internal control in DMSO, data were pulled together and statistical analysis was performed by 1way Anova followed by Tukey post-hoc test (mean and SD, ***p < 0.001, *p < 0.05).

We have previously suggested that the increased levels of LC3-II after LRRK2 kinase inhibition were consequence of an increase in autophagosome production rather than a decrease in degradation. We here corroborate this data by improving the experimental setting including Torin-1 control, evaluating two distinct time-points of treatment, assessing the drug Chloroquine alongside with Bafilomycin (BafA1) and performing co-localization analysis of LAMP1 and p62. As first, H4 cells were co-treated with LRRK2 kinase inhibitor and BafA1 or Chloroquine. Results showed an additive effect of LRRK2in1 over BafA1 (or Chloroquine) treatment alone (Fig. 2a,b, Supplementary Fig. S2a) that appeared at 150 minutes and became significant at 18 hours (Fig. 2c–e), similarly to that observed with Torin-1, an mTOR inhibitor. This confirmed, as previously reported^18^, that the LC3-II increase following inhibition of LRRK2 kinase activity, is different from the effect of BafA1, thus suggesting an increase in macroautophagy flux^34^. Since BafA1, which inhibits autophagosome-lysosome fusion, has an additional, distinct action in preventing lysosomal acidification^35^, the pH of cellular vesicles was assessed in H4 cells treated for 150 minutes or 18 hours with LRRK2in1 or Torin-1, in the presence or absence of BafA1. Using neutral red accumulation no disruption of vesicle acidification was detected either with LRRK2in1 or Torin-1 (Supplementary Fig. S2b), further confirming that the inhibition of LRRK2 kinase activity does not affect lysosomal function. p62 is a cargo protein used to target substrates for degradation through autophagy. We evaluated the co-localization between p62 and LAMP1 (a lysosomal marker) to assess the fusion between lysosomes and autophagosomes^34^,^36^ (Supplementary Fig. S3) showing that, whereas BafA1 was able to reduce the co-localization between p62 and LAMP1 as consequence of inhibition of autophagosome-lysosome fusion, cells treated with Torin-1 or LRRK2in1 were characterized by a higher proportion of co-localized vesicles suggesting that no impairment in autophagy degradation was occurring under such treatments.

### Inhibition of LRRK2 kinase activity induces macroautophagy independently of mTOR/ULK1

**Figure 3:**
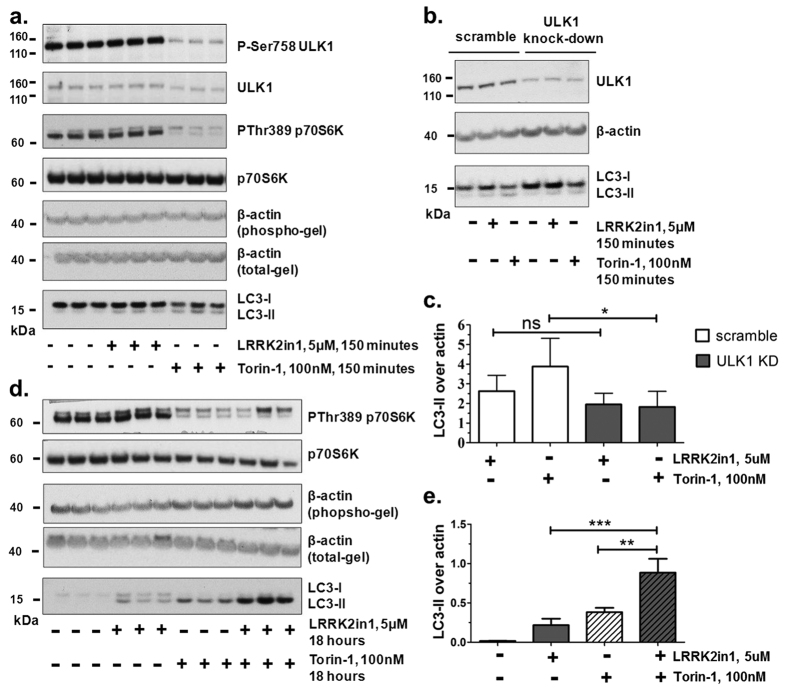
Increased macroautophagy following inhibition of LRRK2 kinase is mTOR independent. (**a**) 150 minutes treatment with LRRK2in1 or Torin-1 (to induce macroautophagy through mTOR). Phosphorylation of P70S6K and ULK1 were used as readout for mTOR inhibition. (**b**) 150 minutes treatment with LRRK2in1 or Torin-1 in scramble controls and in H4 cells with stable ULK1 knock-down (~70% ULK1 knock-down). The gel shown is representative of 4 independent experiments that are quantified in (**c**). (**c**) Quantification was done for each single experiment and LC3-II was normalized against β-actin. After normalization against the control in DMSO, data were pulled together and statistical analysis was performed by 1way Anova followed by Tukey post-hoc test (mean and SD, *p < 0.05). (**d**) 18 hours treatment with LRRK2in1 in the presence and absence of Torin-1. Phosphorylation on P70S6K was used as control for mTOR inhibition. The gel shown is representative of 3 independent experiments each performed in triplicate and quantified in (**e**). (**e**) LC3-II was quantified against β-actin; statistical analysis was performed by 1way Anova followed by Tukey post-hoc test (mean and SD, **p < 0.01, ***p < 0.001).

In our previous work^18^ we did not record any alteration of P70S6K and S6 phosphorylation when macroautophagy was induced by LRRK2 kinase inhibition. This suggested that, in this specific case, induction of macroautophagy may not follow the canonical mTOR pathway. With a new set of tailored experiments we here properly assess that previous suggestion and corroborate the idea that inhibition of LRRK2 kinase activity induces macroautophagy independently of mTOR/ULK1. In canonical macroautophagy, ULK1 is the key downstream effector of mTOR and AMP-activated protein kinase (AMPK) for phagophore generation^37^. Inactivation of mTOR (i.e. following Torin-1 treatment) results in de-phosphorylation of ULK1, and subsequent macroautophagy induction. Canonical macroautophagy induced by Torin-1 treatment and involving mTOR inhibition (as indicated by reduction in the phosphorylation level of p70S6 kinase at Thr389) was strongly associated with decreased mTOR-dependent phosphorylation of ULK1 at Ser758 (Fig. 3a). 150 minutes of LRRK2-in1 treatment was sufficient to increase the levels of the macroautophagy marker LC3-II, however no reduction of ULK1 phosphorylation on Ser758, nor alteration of mTOR activity was observed, as indicated by phosphorylation levels of p70S6K at Thr389 (Fig. 3a).

In order to assess whether ULK1 is required for LRRK2-induced macroautophagy, ULK1 protein levels were stably reduced by 70% in H4 cells transfected with ULK1 shRNA. 150 minute treatment of LRRK2in1 led to a comparable increase of LC3-II production in stable ULK1 knock-down as compared to scrambled control cells. These data indicate that ULK1 is dispensable for macroautophagy induced by LRRK2 kinase activity inhibition (Fig. 3b,c).

Co-treatment with LRRK2in1 and Torin-1 resulted in a stronger increase of LC3-II levels, as compared to single treatments with either LRRK2in1 or Torin-1 (Fig. 3d,e). This data supports an additive effect of the combined inhibition of LRRK2 and mTOR over the macroautophagy flux, further suggesting that these kinases act in parallel pathways to control macroautophagy induction and that the regulation of autophagy by LRRK2 is independent of mTOR/ULK1. Similar results were obtained when m-TOR was alternatively inhibited by aminoacid starvation (Supplementary Fig. S4a,b) or following Rapamycin treatment (Supplementary Fig. S4c), further demonstrating that LRRK2 control over autophagy is independent of mTOR.

### Activation of macroautophagy after inhibition of LRRK2 kinase requires PI3P/Beclin-1

**Figure 4:**
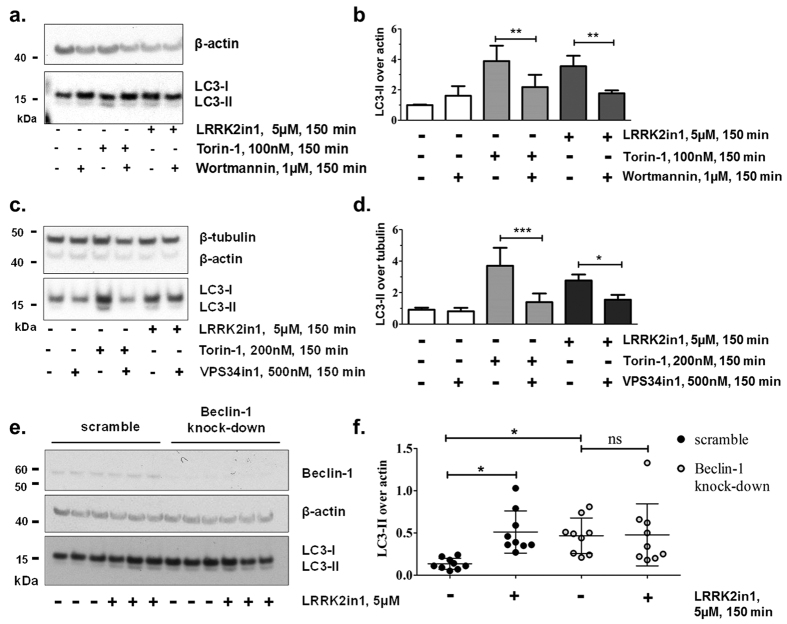
Increase in macroautophagy after inhibition of LRRK2 kinase and Beclin-1. (**a**) 150 minutes treatment with LRRK2in1 or Torin-1 (to induce macroautophagy) in the presence and absence of wortmannin. The gel shown is representative of 3 independent experiments as quantified in (**b**). (**b**) LC3-II was quantified against β-actin; quantification was done for each single experiment; after normalization against the control in DMSO, data were pulled together and statistical analysis was performed by 1way Anova followed by Tukey post-hoc test (mean and SD, ***p < 0.001, **p < 0.01). (**c**) 150 minutes treatment with LRRK2in1 or Torin-1 (to induce macroautophagy) in the presence and absence of VPS34in1. The gel shown is representative of 4 independent experiments as quantified in (**d**). (**d**) LC3-II was quantified against β-actin; quantification was done for each single experiment; after normalization against the control in DMSO, data were pulled together and statistical analysis was performed by 1way Anova followed by Tukey post-hoc test (mean and SD, ***p < 0.001, **p < 0.01). (**e**) 150 minutes treatment with LRRK2in1 in H4 cells stably expressing scrambled shRNA controls or shRNA for Beclin-1. The gel shown is representative of 3 independent experiments, each performed in triplicate and it is quantified in (**f**). (**f**) LC3-II was normalized against β-actin and statistical analysis was performed by Anova followed by Tukey post-hoc test (mean and SD, *p < 0.05).

In addition to the ULK-kinase complex, canonical macroautophagy induction requires the class III PI3-kinase complex containing Beclin-1^38^. WIPI-2 is directly recruited to the nascent autophagosome by the presence of PI3P in the autophagosome membrane, following the activity of the Beclin-1 complex. We have previously shown that LRRK2in1 increases the number of WIPI2 positive punctae in cells^18^. To confirm that this effect can be reproduced, we repeated the prior experiment using a different quantification algorithm (Supplementary Fig. S5). This result suggests that, in contrast to mTOR inhibition and ULK1 activation, PI3P and WIPI-2 may be required for macroautophagy induced by LRRK2 inhibition. To further test this hypothesis, we performed a new and tailored set of experiments. LC3-II levels after LRRK2in1 treatment were assessed in H4 cells treated with wortmannin (or VPS34in1) to inhibit PI3K (or specifically VPS34) and consequently the production of PI3P. Co-treatment with wortmannin (Fig. 4a,b) or VPS34in1 (Fig. 4c,d) and either LRRK2in1 or Torin-1 blocked LRRK2-induced macroautophagy and mTOR-induced macroautophagy respectively further confirming that PI3P is required for autophagosome production under LRRK2 inhibition.

Finally, in order to assess whether Beclin-1 is required for LRRK2-induced macroautophagy, Beclin-1 protein levels were stably reduced in H4 cells transfected with Beclin-1 shRNA(s). Knock-down of Beclin-1 increased the basal level of LC3-II and this was not further increased after 150 minutes treatment with LRRK2-in 1, in contrast to scrambled shRNA controls which showed an enhancement of LC3-II compared to basal levels (Fig. 4e,f). This data further suggested that the macroautophagy pathway controlled by Beclin-1, but not ULK1, is required for the negative regulation by LRRK2 kinase.

## Discussion

Despite its key role in the etiology of a number of human diseases, there is as yet no consensus regarding the physiological function of LRRK2; leading to the suggestion that LRRK2 may have contrasting roles depending on the cell type and condition under investigation^39^. A number of reports have implicated LRRK2 in the regulation of macroautophagy, in endocytosis and metabolism, however the precise molecular function of LRRK2 in these processes is still not defined. The data reported here demonstrate that, at least one of the functions supported by the endogenous LRRK2 kinase activity in a H4 glioma cell line and in primary astrocytes is involved in the non-canonical control of macroautophagy, working parallel to the mTOR/ULK1 pathway and dependent on PI3P and Beclin-1 activity.

The complex events regulating macroautophagy are yet to be fully characterised^40^. Two of the key regulatory systems have, however, been identified: the mTOR/ULK1 and the Beclin-1 pathways. While ULK1 is phosphorylated under basal conditions to repress macroautophagy, ULK1 phosphorylation is lost when macroautophagy flux is induced, via the orchestrated inhibition of mTOR in concert with as yet unidentified phosphatases^41^,^42^. Beclin-1 works in two distinct complexes, both necessary for the production of PI3P that is required first for the formation and later for the maturation of the nascent autophagosome. Non-canonical pathways have been described which do not require proteins crucial for canonical autophagy such as ULK1, Beclin-1 and LC3^43^,^44^,^45^. As an example, the phenotype of the double knock-out for both ULK1 and ULK2 is embryonic lethal, but mice embryonic fibroblasts on this background are able to activate a residual autophagy pathway that is, by definition, independent of ULK1/2 proteins^46^. Moreover, the knock-down of both ULK1 and ULK2 in a B-cell line (DT40) did not alter autophagy^47^, thus confirming that an ULK1/2 alternative route does exist and suggesting different cell types have different ways to control and sustain macroautophagy. Finally, small molecules enhancers of rapamycin (SMERs) have been isolated to induce mTOR-independent autophagy^48^.

In the current study, pharmacological inhibition of LRRK2 kinase activity resulted in an increase of LC3 processing consistent with induction of macroautophagy. Inhibition of mTOR using Torin-1 causes a complete loss of ULK1 phosphorylation on Ser758 and this loss of phosphorylation on ULK1 is required for translocation of the ULK1/2 complex to the pre-autophagosomal structure^37^. In contrast, the lack of alteration in ULK1 phosphorylation following LRRK2 kinase inhibition, suggests the possible activation of a non-canonical pathway independent of ULK1. Autophagosomes produced due to loss of LRRK2 kinase activity were positive for WIPI-2 thus suggesting the activity of Beclin-1 and the generation of PI3P was conserved. Indeed, the inhibition of PI3P production by wortmannin or VPS34in1 was able to inhibit the induction of LRRK2-regulated macroautophagy. Finally, Beclin-1 knock-down cells were not responsive to LRRK2in1 treatment, thus confirming that Beclin-1/PI3P are involved either upstream or downstream of the induction of macroautophagy via LRRK2 inhibition. A possible link between Beclin-1 and LRRK2 is intriguing given the existing literature. The G2019S-LRRK2 mutant (which shows increased kinase activity), is able to bind to and phosphorylate Bcl-2 resulting in dysregulation of mitophagy^49^. Beclin-1 contains Bcl-2 binding sites that are important for its macroautophagy regulatory activities^50^. Beclin-1 has been found to be phosphorylated and regulated by the stress responsive kinases MAPKAPK2 and 3^51^; LRRK2 has been suggested to bind and phosphorylate MAP2K3 (MKK3) upstream of MAPKAPK2/3 in the p38-MAPK pathway^52^. It is also of note that the Beclin complex has a complex role in the regulation of macroautophagy, dependent upon the binding partners Beclin-1 is associated with^53^,^54^,^55^. In the presence of ATG14L or UVRAG, Beclin-1 acts as a positive regulator of macroautophagy – consistent with the data presented herein from H4 cells. In contrast, when bound to RUBICON Beclin-1 acts as a repressor of autophagy. Clarifying how LRRK2 relates to these distinct Beclin complexes will provide further insight into the precise mechanisms whereby LRRK2 can impact on autophagy, and may provide an explanation for the divergence in experimental data across the research literature. Strikingly, regulation of macroautophagy by LRRK2 is echoed by studies on Death Associated Protein kinase 1 (DAPK1), another member of the ROCO protein family to which LRRK2 belongs, and that has been proposed to be involved in the control of macroautophagy by both direct and indirect phosphorylation of Beclin-1^56^,^57^. Members of the ROCO family share a high homology in their GTPase and COR domains, while the kinase domains of LRRK2 and DAPK1 belong to different families. There is accumulating evidence, however, that the kinase and the GTPase activities present in LRRK2 are able to regulate one another^58^. These observations suggest that an understanding of both the kinase and GTPase activities of LRRK2 may be required to fully illuminate LRRK2’s role in the regulation of macroautophagy. Within the current study we make use of chemical inhibition of LRRK2 kinase activity to infer how the endogenous protein regulates macroautophagy by physiological signaling in cells. However, we cannot currently determine if the effects here are solely due to kinase activity or are partially mediated through, for example, the GTPase function of LRRK2. Further experiments addressing the GTPase activity of LRRK2, either through genetic manipulations or small molecules that target this region of the protein, are therefore important in the future.The close genetic ties between LRRK2 and PD have resulted in extensive efforts to understand LRRK2 in the context of this disorder. The recent discovery of significant links between LRRK2 and a range of other, apparently unrelated, human disorders has further emphasised the importance of this protein to human health. A major challenge for LRRK2 research is, therefore, to reconcile the involvement of LRRK2 in the pathological pathways underlying these disparate disorders. Several complementary strands of evidence suggest that one key physiological function of LRRK2 is with regard to the control of macroautophagy and vesicle dynamics, raising the possibility that this may be the common theme behind the different disorders in the pathogenesis of which LRRK2 has been implicated. Another major problem in the study of LRRK2 patho-physiology is represented by the fact that it has been associated with a multiple array of cellular functions^59^, and has many protein interactors^60^, suggesting that LRRK2 acts as a hub protein able to work with a wide range of partners. This has led to the suggestion that LRRK2 may have different roles in different cell types and its function may be different depending on the situation/stimuli. The data presented in this study provides new insight into the dissection of the mechanism of LRRK2 function, information that will be critical for understanding the connection between LRRK2 and disease pathogenesis, to provide the basis for therapeutic intervention directed at LRRK2s activities and to consider the side effects and safety issues of chronic LRRK2 kinase inhibition in a human context.

## Materials and Methods

### Reagents

The LRRK2in1 compound and the VPS34in1 were purchased from the Division of Signal Transduction Therapy, School of Life Sciences, and University of Dundee, UK. The GSK2578215A compound was purchased from Tocris. Bafilomycin A1 (B1793-2UG), Chloroquine (C6628) and Wortmannin (W3144-250UL) were purchased from Sigma-Aldrich. Torin-1 (CAY10997) was purchased from Cayman Chemicals.

Antibodies used were as follows: LC3 antibody (NB100-2220, Novus Biologicals); LRRK2 antibodies (MJFF#2, 3514-1/ab133474, Epitomics); total S6 antibody (2317, Cell Signalling); phospho Ser235/236 S6 antibody (2211S, Cell Signalling); total P70S6K antibody (sc-8418, Santa Cruz); phospho Thr389 P70S6K (sc-11759, Santa Cruz); total ULK1 antibody (8054 and 4773, Cell Signalling); phospho Ser757 ULK1 antibody (6888, Cell Signalling); Beclin-1 antibody (3738, Cell Signalling); β-actin antibody (A1978, Sigma Aldrich); β-tubulin (T6199, Sigma Aldrich).

### H4 cell culture and treatment

H4 cells (ATCC number HTB-148) were grown in DMEM containing 10% FCS. After 24 hours from plating, when at 80% confluence, H4 cells were treated with LRRK2 inhibitors LRRK2in1 or GSK2578215A, with BafA1 or Torin-1 or wortmannin at the concentrations and for the time reported in each experiment. For each experiment, DMSO vehicle controls were added. After washing in Dulbecco’s phosphate buffered saline (DPBS) cells were collected in a lysis buffer containing: 0.5% Triton X-100, 2 mM ethylen di-ammonium tetra acetic acid (EDTA), 150 mM NaCl, 0.5% sodium deoxycholate, 0.1% sodium dodecyl sulphate (SDS), protease inhibitors (cOmplete, protease inhibitor cocktail, Roche) and phosphatase inhibitors (Halt phosphatase inhibitor cocktail, Pierce) in 50 mM TRIS-HCl pH 7.5.

### Primary astrocytes culture

Primary astrocyte cultures were prepared from P3-4 mouse forebrain following a protocol adapted from Schildge *et al*.^61^. Briefly, brains were isolated in cold HBSS (Sigma-Aldrich). The forebrains were dissected and meninges removed. Forebrains were dissociated before incubation in papain (Worthington) at 37 °C for 30 min. The samples were triturated by pipetting and subsequently plated in DMEM supplemented with 10% FCS and 1% penicillin/streptomycin. At 14 divisions, microglia were dissociated by agitation for 1 hr at room temperature and astrocytes were re-plated following brief trypsinization to expand the culture. Astrocytes were aged to 45/47 divisions, plated in 6 well plates (1 × 10^6^ cells/well), and at 80% confluency the cells were treated with LRRK2in1 or DMSO (control) as reported in figures. Following treatment, the cells were washed in DPBS, collected by scraping and cell pellets were frozen until Western blot analysis.

### Western blotting

Cell lysates were frozen immediately upon collection or kept at 4 °C for 30 minutes; following thawing, they were clarified by centrifugation at 13000 rpm for 5 minutes at 4 °C, protein concentration was assessed by BCA assay (BCA Protein Assay Kit, Pierce) and 10–15 μg aliquots were added of NuPAGE sample buffer containing 2-mercaptoethanol (Invitrogen), denatured for 10 minutes at 70 °C and analysed using NuPAGE, Novex precasted Bis-Tris 4–12% (Invitrogen), in MES running buffer (Invitrogen). After electrophoresis, gels were blotted onto 0.45 μm cut-off, PVDF through conventional blotting. Membranes were blocked and processed with peroxidase-conjugated antibodies for enhanced chemiluminescence (ECL) detection. Films were acquired as images in jpg format using an EPSON Perfection 4870 photo scanner and processed by the ImageJ software ( http://rsbweb.nih.gov/ij/).

### Statistics

All the results have been repeated in at least 3 independent experiments (details are given in each figure legend). In the case of Western blot analysis for phospho-proteins, samples were divided in half and run in two parallel gels; one was processed for the phospho-antibody and one for the total-antibody (to avoid membrane stripping and re-probing). The total and the phospho bands were normalized against the respective β-actin loading control; then, the ratio of (normalized)phospho over (normalized)total protein was calculated. Statistical analyses were performed by the use of the Prism software (GraphPad) as described in each figure legend.

### Generation of stable knock-down

H4 cells were transfected with 2 to 10 μg LRRK2 shRNA, ULK1 shRNA, Beclin-1 shRNA or scramble Open Biosystems GIPZ shRNAmir (V3LHS-644167, V2LHS-33057, V3LHS-349512 and -349514, Thermo Fisher Scientific) using PEI (Sigma-Aldrich) transfection reagent according to the manufacturer’s instructions. ShRNA vectors contain a puromycin resistance gene. Cells were treated with 2 μg/ml puromycin supplemented DMEM 48 hrs after transfection and kept under selection for expansion. Puromycin selection was removed 24 hours before the experiment to avoid interference of the antibiotic with the treatment.

## Additional Information

**How to cite this article**: Manzoni, C. *et al*. mTOR independent regulation of macroautophagy by Leucine Rich Repeat Kinase 2 via Beclin-1. *Sci. Rep.* **6**, 35106; doi: 10.1038/srep35106 (2016).

## Supplementary Material

Supplementary Information
