# Supplementary material for: mTOR independent regulation of macroautophagy by Leucine Rich Repeat Kinase 2 via Beclin-1
Source: Sci Rep. 2016 Oct 12;6:35106. doi: 10.1038/srep35106 (PMC5059726; doi:10.1038/srep35106)
Supplement: Supplementary Information [file srep35106-s1.pdf]

mTOR independent regulation of macroautophagy by Leucine Rich Repeat Kinase 2 via Beclin-1

Claudia Manzoni[1, 2]*, Adamantios Mamais[3], Dorien Roosen[3], Sybille Dihanich[2], Marc Soutar[2], Helene Plun-Favreau[2], Rina Bandopadhyay[4], John Hardy[2], Sharon A. Tooze[5] , Mark R. Cookson[3] and Patrick A. Lewis[1, 2]*

1. School of Pharmacy, University of Reading, Whiteknights, Reading, RG6 6AP, United Kingdom

2. Department of Molecular Neuroscience, UCL Institute of Neurology, Queen Square, London WC1N 3BG, United Kingdom

3. Laboratory of Neurogenetics, NIH, Bldg. 35 9000 Rockville Pike, Bethesda, MD 20892, USA

4. Reta Lila Weston Institute of Neurological Studies, UCL Institute of Neurology, 1 Wakefield Street London WC1N 1PJ, United Kingdom

5. Francis Crick Institute, Lincoln's Inn Fields Laboratories, 44 Lincoln's Inn Fields London, WC2A 3LY, United Kingdom

*To whom correspondence should be addressed: c.manzoni@reading.ac.uk *or* p.a.lewis@reading.ac.uk

Running Title

Macroautophagy is regulated by LRRK2 *via* Beclin-1

Figure S1

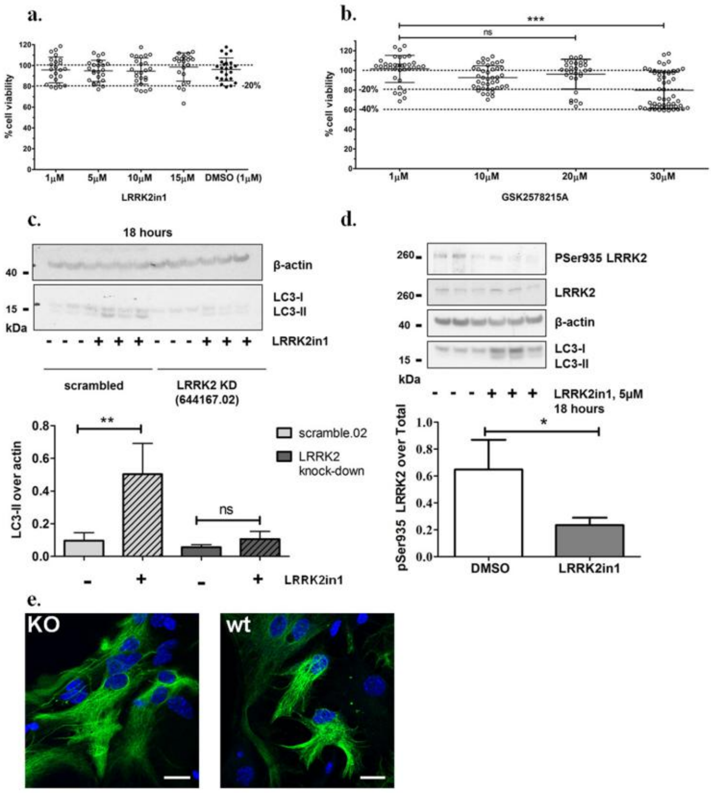

Figure S1

Additional controls for inhibition of LRRK2 kinase leading to increase in LC3-II. (a) LRRK2in1 and (b) GSK2578215A toxicity evaluation performed by MTT assay (Sigma-Aldrich, M2128) according to the supplier instructions. Data were collected in 3 independent experiments and each single dosage was normalized to its own DMSO control; statistical analysis was performed by 1way Anova followed by Dunnett's post-hoc test. (c) 18 hours LRRK2in-1 treatment in scramble controls and in LRRK2 stable knock-down H4 cells; the image shown is representative of 3 independent experiments and it is quantified in the graph; LC3-II was quantified against β-actin; statistical analysis was performed by 1way Anova followed by Tukey post-hoc test (mean and SD, ** = $p<0.01$). **(**d) Reduction of LRRK2 phosphorylation on Ser935 after 18 hours LRRK2in-1 treatment, student t-test (mean and SD, pvalue * = 0.0350). (e) Primary astrocytes from LRRK2 knock-out mice (KO) and wild-type controls (wt) stained for glia fibrillary acidic protein (GFAP, in green), to evaluate enrichment of astrocytes over other potential contaminant cell types; nuclei are stained with Hoechst (depicted in blue); scale bar = 20µm.

Figure S2

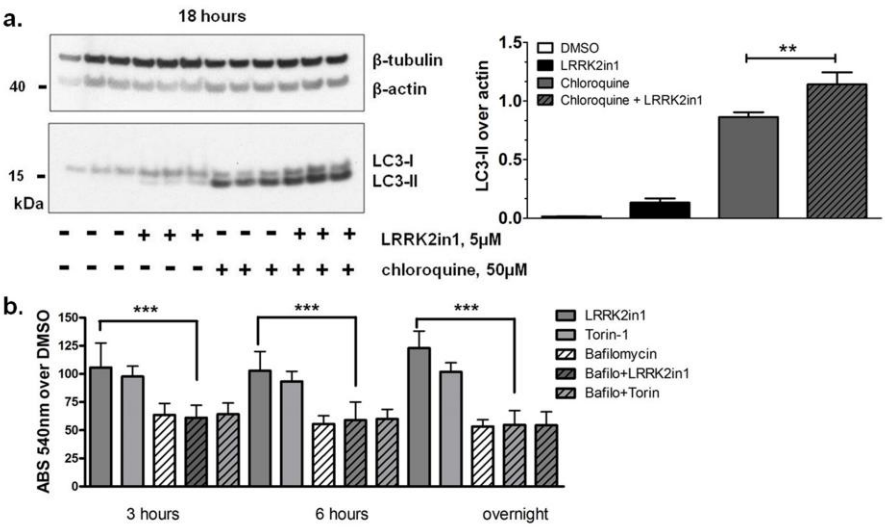

Figure S2

Additional controls for inhibition of LRRK2 kinase leading to increase in autophagy flux. (a) 18 hours treatment with LRRK2in-1 in the presence and absence of Chloroquine to block lysosomal acidification. The gel shown is representative of 3 independent experiments, each performed in triplicate and it is quantified in the graph; LC3-II is quantified against β-tubulin; statistical analysis was performed by 1way Anova followed by Tukey post-hoc test (mean and SD, ** = $p<0.01$).

(b) Neutral Red staining: Cells were plated in 96 well plates and treated with DMSO, LRRK2in1, Torin-1 and/or BafA1. Following treatment, cell culture medium was added of 3-amino-7-dimethylamino-2-methyl-phenazine hydrochloride (NR = neutral red, Sigma Aldrich) to reach the final concentration of 80 ng/ml; the dye was left in the cell culture medium for 30 mins. Cells were washed twice and lysed in 50% ethanol, 49% deionized water, 1% glacial acetic acid. Absorbance was recorded by a multiwell plate reader at the wavelength of 540 nm. Data were expressed as percentage of neutral red staining normalized to untreated controls. 3, 6 hours and overnight treatment with LRRK2in-1 or Torin-1 (to induce the autophagy flux) in the presence and absence of BafA1 (to block lysosomal acidification). Cells have been exposed to the neutral red dye to stain and quantify acidic vesicles/lysosomes. The experiment was independently replicated 2 times, with 6 to 12 technical replicates for each condition in each independent experiment. The change in NR staining was normalized against the control in DMSO, statistical analysis was performed by Anova followed by Tukey post-hoc test. (mean and SD, *** = $p<0.001$).

Figure S3

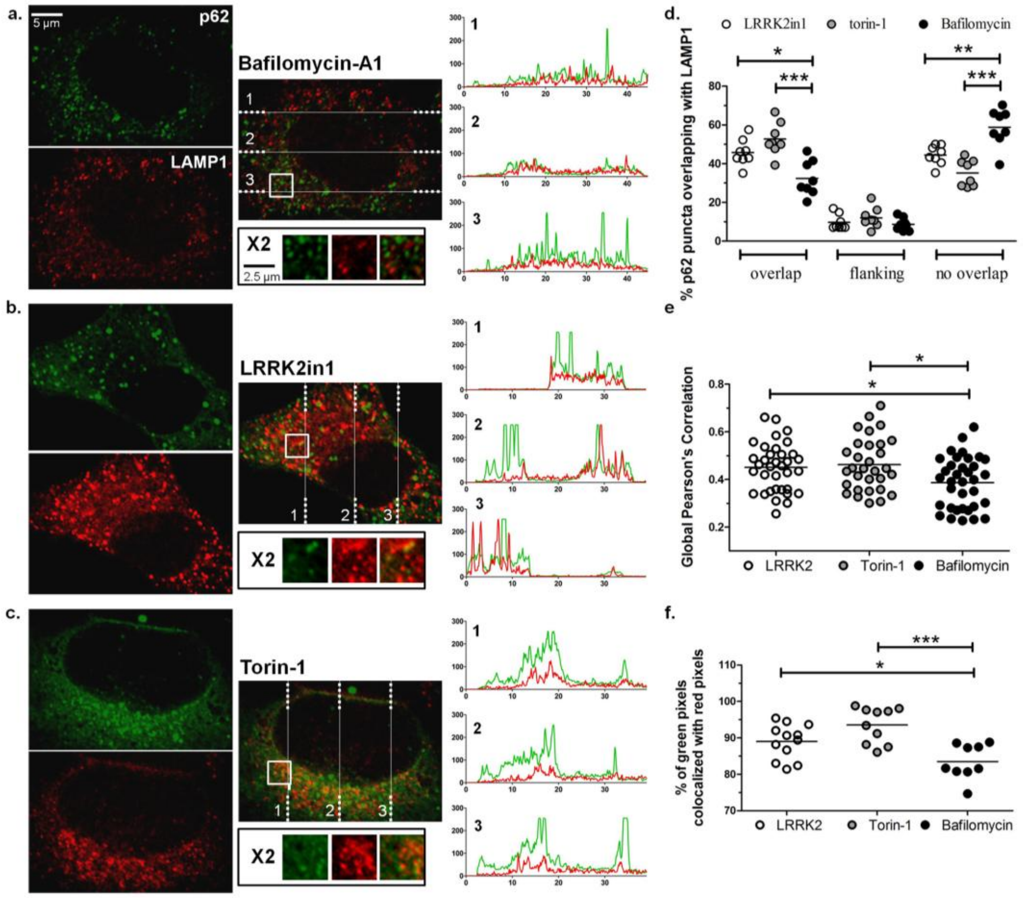

Figure S3

Co-localization of p62 and LAMP1.

H4 cells were seeded on coverslips. At the end of the overnight treatment [(a)Baf-A1 40nM, (b)LRRK2in1 5µM and (c)Torin-1 100nM] cells were washed twice in DPBS and fixed at room temperature for 15 minutes in a solution of 4% paraformaldehyde in DPBS. Cells were washed three times in DPBS, blocked and permeabilized at room temperature with 0.1% Triton X-100 in 15% normal goat serum (S1000, Vector) DPBS. Cells were incubated overnight at 4 ◦C with the primary antibody (mouse LAMP1; SantaCruz and rabbit p62/SQSTM1, Enzo). Anti-mouse, (Alexa Fluor, emission at 568 nm) and anti-rabbit, (Alexa Fluor, emission at 488 nm) were used as secondary antibodies and nuclei were labelled with Hoechst 33342. Coverslips were sealed with Fluoromount G mounting medium (Southern Biotech) and images were acquired with 63X magnification with a Zeiss LSM 710 confocal microscope and processed by the Zen 2009 software. Three independent experiments were run, for each of them multiple images were acquired from different coverslips as follows: exp#1: 1 coverslip for a total of 3 images acquired for each treatment; exp#2: 2 different coverslips for a total of 6 images acquired for each treatment; exp#3: 2 different coverslips for a total

of 5 images acquired for each treatment. Scale bar = 5µm; the insets report a two-folds magnification, scale bar = 2.5µm. Evaluation of green(p62)-red(LAMP1) co-localization was performed with 3 different approaches. (d) The profile plot of 8 different pictures (28 Baf-A1, 18 Torin-1 and 25 LRRK2in1 treated cells) was calculated by the Zen 2009 software after dividing the picture in 10/20 horizontal frames (depending on the number of cells in frame). For each frame the profile plot was acquired and scored manually by a operator considering the number of green peaks overlapping/not-overlapping/flanking red peaks. The score of each frame was combined to obtained the final score of the total image and results were calculated as percentage over the total amount of green peaks per image. Single data point are reported in the graph, statistical analysis was performed by 1way ANOVA followed by Tukey's post-hoc test. (e) Images were processed through the Volocity (PerkinElmer) image processing software for a total of 35 Baf-A1, 31 Torin-1 and 35 LRRK2in1 treated cells. Each cell was selected and the Global Pearson's Correlation Coefficient was calculated between the green and the red channels. Single data point are reported in the graph, statistical analysis was performed by 1way ANOVA followed by Tukey's post-hoc test. (f) the plot profiles acquired as in (a) (9 plots for Baf-A1; 10 plots for Torin-1 and 12 plots for LRRK2in1) were saved as .txt files and processed through an in-house R script to automatically calculate the number of overlapping green/red pixels. A threshold (T) was applied to remove the background noise. For each single pixel the relative intensity on the red vs green channels was calculated and a second threshold (W) was applied to distinguish between overlapping pixels (above W) from not-overlapping pixels (below W). Both T and W were set up in order to maximize the difference between the positive (Torin-1) and the negative (Baf-A1) controls. The number of pixels single plots within an image were then combine to obtain the final score for the total image processed. The results were expressed as percentage of co-localized pixel over the total amount of green pixels in the image. Single data point are reported in the graph, statistical analysis was performed by 1way ANOVA followed by Tukey's post-hoc test (* = $p<0.05$, ** = $p<0.01$, *** = $p<0.001$).

Figure S4

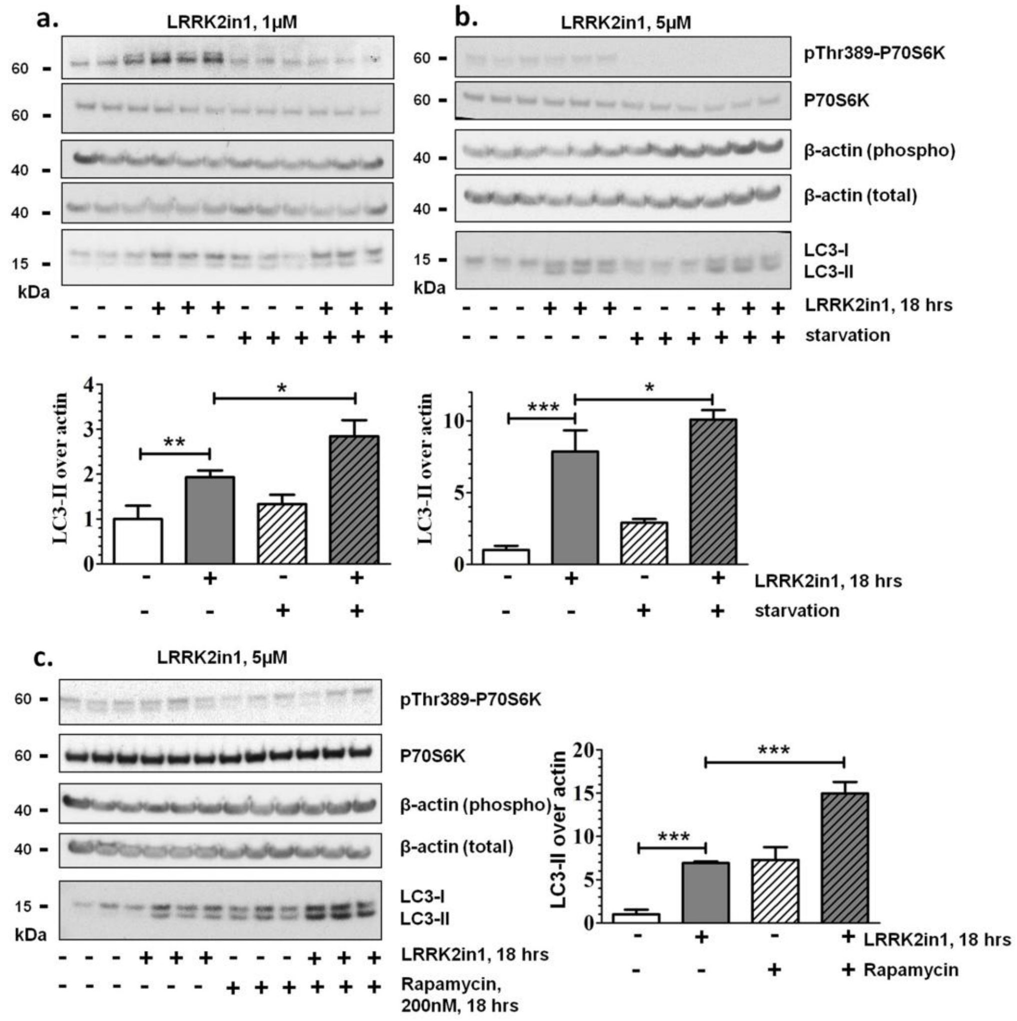

Figure S4

Additional controls for mTOR inhibition (starvation and Rapamycin treatment)

18 hours treatment with (a) LRRK2in-1, 1µM or (b) LRRK2, 5µM in the presence and absence of aminoacid starvation (overnight starvation from serum, followed by 150 minutes starvation from aminoacids). Phosphorylation on P70S6K was used as control for mTOR inhibition. The gel shown is representative of 3 independent experiments each performed in triplicate and quantified in the graph. LC3-II was quantified against β-actin; statistical analysis was performed by 1way Anova followed by Tukey post-hoc test. (c) 18 hours treatment with 5µM LRRK2 in the presence and absence of Rapamycin. Phosphorylation on P70S6K was used as control for mTOR inhibition. The gel shown is representative of 3 independent experiments each performed in triplicate and quantified in the graph. LC3-II was quantified against β-actin; statistical analysis was performed by 1way Anova followed by Tukey post-hoc test (mean and SD, ** = p<0.01, *** = p<0.001).

Figure S5

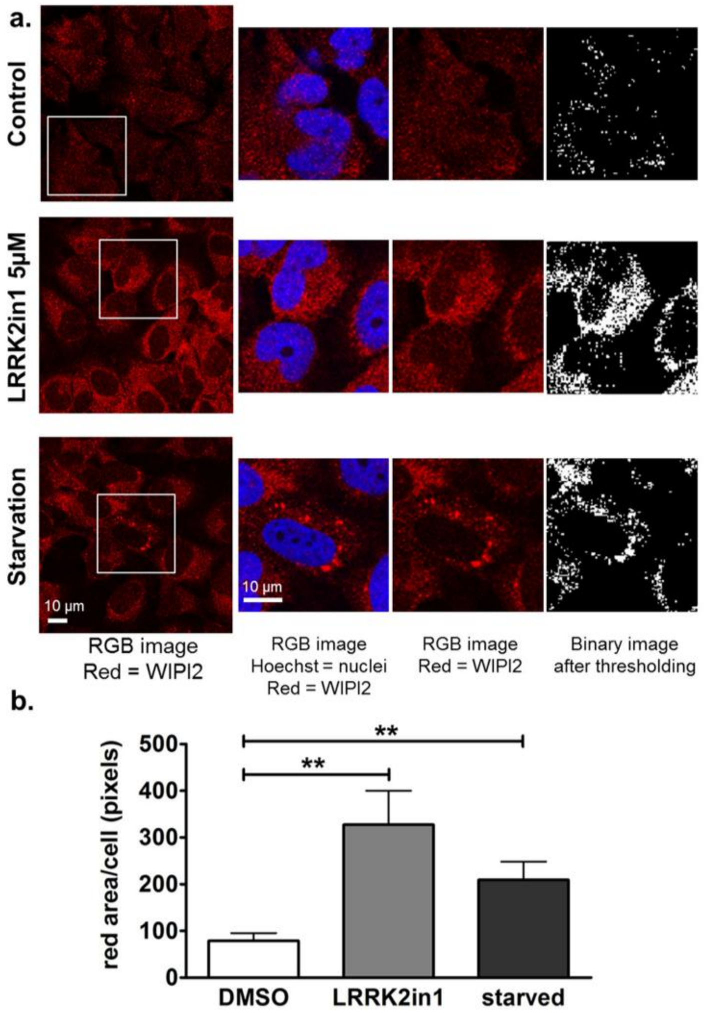

Figure S5

Increase in macroautophagy after inhibition of LRRK2 kinase requires PI3P production. (a) WIPI2 (red) and Hoechst (blue) staining after 18 hours treatment with LRRK2in1 or after starvation (16 hours without serum followed by 3 hours amino acid starvation to induce macroautophagy). Cells were seeded on coverslips. At the end of the treatment (DMSO only, starvation and LRRK2in1) cells were washed twice in DPBS and fixed at room temperature for 15 minutes in a solution of 4% paraformaldehyde in DPBS. Cells were washed three times in DPBS, blocked and permeabilized at room temperature with 0.1% Triton X-100 in 15% normal goat serum (S1000, Vector) DPBS. Cells were incubated overnight at 4 ◦C with the primary antibody (mouse WIPI2 antibody, kindly supplied by S.Tooze). Anti-mouse, (Alexa Fluor, emission at 568 nm) was used as secondary antibody and nuclei were labelled with Hoechst 33342. Coverslips were sealed with Fluoromount G mounting medium (Southern Biotech) and images were acquired with a Zeiss LSM 710 confocal microscope and processed by the Zen 2009 software. Images were processed as follows: Confocal images were saved as JPG files and automatically processed with a custom made image analysis program

(cell.count()) run in Octave (version 3.8.0, GNU General Public Licence, https://www.gnu.org/software/octave/), installed libraries: GNU plot, Image. Briefly, each single image was opened and converted from RGB to grey scale. Threshold has been manually set at “20” and all they grey spot above the threshold were automatically counted to calculate the total amount of WIPI2 positive pixels in the picture. The script is appended as Supplementary Dataset S1 online. Briefly, after thresholding, for each picture (frame) the area (pixel) covered by the staining was quantified and normalized to the number of nuclei in the frame. Three independent experiments were performed; for a total of 7, 7 and 6 independent slides stained for DMSO, LRRK2in-1 and starved controls respectively; each single slide was sampled 3 times (the final numbers of quantified frames are: n= 21 for DMSO, n= 21 for LRRK2in-1, n= 18 for starved controls). b) Mean and SEM are reported in graph; statistical analysis was performed by student t-test (pvalue DMSO vs LRRK2in-1 = 0.0018; pvalue DMSO vs starved = 0.0026).

Supplementary Dataset S1

Imaging Analysis

```
function cell.count()

clc; close all; clear all;
pkg load all;

cd 'Input'
ImageName = 'input the name of the picture to be processed';
img = imread (ImageName);
figure (1);
imshow(img);
cd ..

folder = ['Output-' ImageName];
confirm_recursive_rmdir(false);
rmdir(folder,'s');
mkdir(folder);

cd (folder)
setenv('GSC','GSC');
print Original.png
cd ..

Grey = rgb2gray(img);
figure(2);
imshow(Grey);

cd (folder);
setenv('GSC','GSC');
print Grey.png
cd ..

% pixel above treshold are white and below are black
Grey = thresholdImg(Grey,20);
figure(3);
imshow(Grey);

cd (folder)
setenv('GSC','GSC');
print GreyTreshHold.png
cd ..

Grey_mod = logical(Grey/255);
```

```
object = bwlabel(Grey_mod,8);
figure(4)
imshow(object)

n = max(max(object))
white = sum(sum(Grey/255))

cd (folder)
setenv('GSC','GSC');
print GreyObjects.png
cd ..
```
